# Design of Deep Eutectic Systems: A Simple Approach for Preselecting Eutectic Mixture Constituents

**DOI:** 10.3390/molecules25051077

**Published:** 2020-02-28

**Authors:** Ahmad Alhadid, Liudmila Mokrushina, Mirjana Minceva

**Affiliations:** 1 Biothermodynamics, TUM School of Life Sciences Weihenstephan, Technical University of Munich, Maximus-von-Imhof-Forum 2, 85354 Freising, Germany; 2 Separation Science & Technology, Friedrich-Alexander-Universität Erlangen-Nürnberg (FAU), Egerlandstr. 3, 91058 Erlangen, Germany

**Keywords:** eutectic mixtures, deep eutectic solvents, solid–liquid equilibria, hydrophobic DESs, melting properties

## Abstract

Eutectic systems offer a wide range of new (green) designer solvents for diverse applications. However, due to the large pool of possible compounds, selecting compounds that form eutectic systems is not straightforward. In this study, a simple approach for preselecting possible candidates from a pool of substances sharing the same chemical functionality was presented. First, the melting entropy of single compounds was correlated with their molecular structure to calculate their melting enthalpy. Subsequently, the eutectic temperature of the screened binary systems was qualitatively predicted, and the systems were ordered according to the depth of the eutectic temperature. The approach was demonstrated for six hydrophobic eutectic systems composed of L-menthol and monocarboxylic acids with linear and cyclic structures. It was found that the melting entropy of compounds sharing the same functionality could be well correlated with their molecular structures. As a result, when the two acids had a similar melting temperature, the melting enthalpy of a rigid acid was found to be lower than that of a flexible acid. It was demonstrated that compounds with more rigid molecular structures could form deeper eutectics. The proposed approach could decrease the experimental efforts required to design deep eutectic solvents, particularly when the melting enthalpy of pure components is not available.

## 1. Introduction

Eutectic systems are mixtures of two or more compounds that exhibit partial immiscibility or negligible mutual solubility in the solid phase [1]. Deep eutectic solvents (DESs) are eutectic mixtures characterized by a large depression of the melting temperature of the mixture at the eutectic point relative to the melting temperature of the pure components [2,3]. DESs are analogous to ionic liquids (ILs) in terms of being designer solvents and possessing low vapor pressure. However, DESs are usually less toxic, easier to prepare, and less expensive than ILs. These advantages have led to the recent increase in applications of DESs, for example, as solvents in diverse separation methods [4,5,6,7,8], media for chemical [9,10,11,12,13,14], electrochemical [15,16,17,18,19], and biological reactions [20,21], in polymer chemistry [22,23,24], and for increasing the solubility of active pharmaceutical ingredients [25,26,27,28].

Although their preparation may be easier than that of ILs, DESs are more difficult to design. The ratio of the ions in ILs is defined by the electroneutrality of the solution. In contrast, the ratio of DES components is not fixed and can be of any value. One of the first pieces of information required when designing DESs for a specific application is the eutectic temperature and eutectic composition of the system. Thus far, the design of DESs has been performed primarily using a trial and error approach. In most published works, preselected components are mixed at several fixed molar ratios, such as 1:1 or 1:2, and mixtures that remain liquid at room temperature are selected for further testing [29,30]. To determine the system composition and melting temperature at the eutectic point, the solid–liquid equilibria (SLE) of the eutectic systems should be known. The SLE also provides information about the melting temperature of the system at any specific composition.

The experimental determination of SLE phase diagrams of eutectic systems is non-trivial and is often accompanied by difficulties and limitations. For example, the hygroscopic nature of some DES components [31], the high viscosity and paste-like consistency of some DESs close to their melting temperature [32], the decomposition of DES constituents before melting, and the chemical reaction between DES constituents after storage [33]. Owing to the previously mentioned difficulties, predictive methods are required. Abranches et al. [34] proposed Conductor like Screening Model for Real Solvents (COSMO-RS) to predict the SLE of eutectic mixtures. Wolbert et al. [35] used UNIFAC (Do) to model the activity coefficient of constituents in binary eutectic mixtures.

The SLE of simple eutectic systems, whose components show negligible mutual solubility in the solid phase, is commonly calculated in the literature using the following simplified equation:

(1) $$\ln x_{i}^{L}\gamma_{i}^{L}=\frac{∆h_{m,i}}{R}\left(\frac{1}{T_{m,i}}-\frac{1}{T}\right)$$

where $x_{i}^{L}\;$and $\gamma_{i}^{L}$ are the mole fraction and activity coefficient of the component i in the liquid solution, respectively; $∆h_{m,i}$ and $T_{m,i}$ are the melting enthalpy and melting temperature of the pure component i, respectively; T is the liquidus temperature (i.e., melting temperature of the mixture at the mole fraction $x_{i}^{L}$); and R is the universal gas constant. As seen in Equation (1), SLE calculations require information about the pure components melting properties, namely, the melting enthalpy $∆h_{m,i}$ and temperature $T_{m,i}$, as well as information about the behavior of the components in the liquid phase (i.e., activity coefficients$\;\gamma_{i}^{L}$).

Strong intermolecular interactions between unlike molecules in the liquid phase—low activity coefficient values of components—and/or low melting enthalpy values of pure components lead to a deep depression of the melting temperature of the mixture at the eutectic point [35,36,37,38]. The melting enthalpy of components is not always easily measured because of polymorphism, kinetic limitations, and/or thermal instability. As a result, the melting enthalpy of many components is unavailable. The aim of this work was to demonstrate the correlation between the molecular structure and melting enthalpy of a component to simplify the selection of components, especially when no experimental data on melting enthalpy is available.

At the melting point of a pure component, the solid and liquid phases are in equilibrium. The melting temperature $T_{m}$ of a pure component is the ratio between the melting enthalpy $∆h_{m}$ and the melting entropy $∆s_{m}$:

(2) $$T_{m}=\;\frac{∆h_{m}}{∆s_{m}}$$

Melting enthalpy is the energy required to melt solid crystals [39] and depends on the type of interactions between molecules in the lattice structure [39,40]. Melting entropy is the increase in the disorder and randomness upon melting [41] and depends on the molecular symmetry and conformational degrees of freedom of the molecule [41,42,43,44]. Despite several attempts to correlate the melting properties of a component with its molecular structure, there is no generic model that can predict the melting properties of pure components [45].

The melting temperature of pure components is difficult to predict [43]; however, unlike the melting enthalpy, experimental data on the melting temperature are, in many cases, available. However, depending on the method of determination as well as the purity of the components, the reported values may deviate by several degrees Celsius from the actual melting temperature. However, as seen from Equation (1), an uncertainty of several degrees Celsius in the melting temperature of pure components would have a small effect on the SLE of the mixture. According to Bondi [46], the melting entropy of compounds can be better related to the molecular structure than the melting enthalpy. The melting entropy calculated from the molecular structure with the available melting temperature can be used to estimate the melting enthalpy of the components with Equation (2). Using this information, the eutectic temperature can be approximately calculated using Equation (1) under the assumption of ideal behavior$\;(\gamma_{i}^{L}=1$).

The objective of this study was to test a simple approach that could be used to select potential eutectic system constituents based on their melting enthalpies. It is assumed that mixtures of components with a lower melting enthalpy would result in eutectic mixtures with a larger melting temperature depression, as previously demonstrated [35,36,37,38]. This approach aims to reduce the experimental efforts required to measure pure components’ melting enthalpy, as well as the SLE of eutectic mixtures. To evaluate the proposed approach, binary eutectic mixtures of L-menthol with six different monocarboxylic acids were considered in this work. The goal was to predict the eutectic temperature of each system relative to other systems. Although the eutectic temperatures were calculated with the unitary activity coefficient, it was not claimed that the components should behave ideally. The proposed approach was based on the assumption that any component in its binary solutions with other components sharing the same type and number of functional groups behaves similarly (i.e., in any binary mixture with monocarboxylic acids, L-menthol behaves in a similar manner). The latter assumption has been validated for many eutectic systems, for example, in [Ch]Cl/sugar [32], [Ch]Cl/dicarboxylic acids [47], [Ch]Cl/fatty acids or alcohols [48], and thymol/fatty acids [49].

## 2. Results and Discussion

### 2.1. Melting Properties of Pure Components

The melting entropy of acids was calculated using the model proposed by Jain et al. [44] (Equations (4)–(6)). The model parameters and calculated melting entropies are presented in Table 1. Linear acids possessed higher melting entropy than cyclic acids. This was a result of the higher flexibility of a linear chain compared to a ring structure. The lowest predicted melting entropy was for cyclohexanecarboxylic acid with a flexibility number Φ equal to zero. The model predicted the same melting entropy for 3-phenylpropionic acid and 3-cyclohexylpropionic acid. This was because the model did not differentiate between phenyl and aliphatic ring and predicted the same flexibility number Φ for both components. Due to the asymmetry of the carboxylic acid group, the symmetry number σ of all acids tested in this work was one.

Table 2 presents the melting properties that were experimentally determined in this study using DSC. The obtained values were in good agreement with those reported in the literature. To the best of our knowledge, the melting properties of cyclohexylpropionic acid have not been measured before. As seen in Table 2, the melting enthalpy of l-menthol had a low value, thus making l-menthol a good candidate for designing deep eutectic systems. In general, linear acids have higher melting enthalpies than cyclic acids. The melting enthalpy of linear acids increases by increasing the chain length; for example, lauric acid > capric acid > caprylic acid. The lowest melting enthalpy was observed for cyclohexanecarboxylic acid, which is the component with the most rigid molecular structure.

Table 3 presents a comparison between the melting entropies predicted by the model by Jain et al. [44] (Equations (4)–(6)) and the experimental melting entropies calculated with Equation (2) using the experimentally determined melting enthalpy and melting temperatures of acids. As seen in Table 3, the predicted melting entropy of all acids was overestimated.

**Figure 1:**
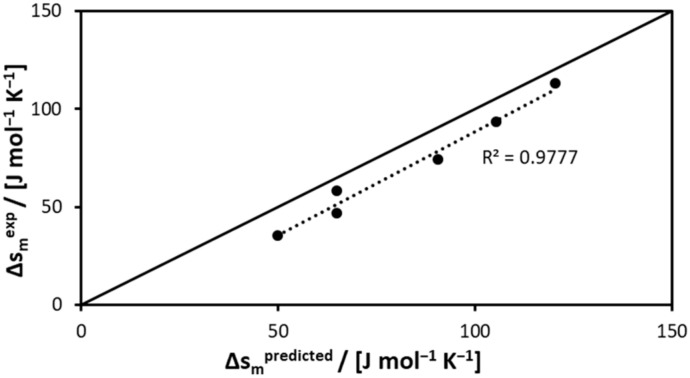
Experimental melting entropies measured in this study in comparison with predicted melting entropies.

Figure 1 depicts the predicted melting entropy values in comparison to the experimental values. The linear correlation between the predicted melting entropies indicated that the model proposed by Jain et al. [44] could provide a reasonably good estimation of the melting entropy of compounds sharing the same chemical functionality.

### 2.2. Solid–Liquid Equilibria

**Figure 2:**
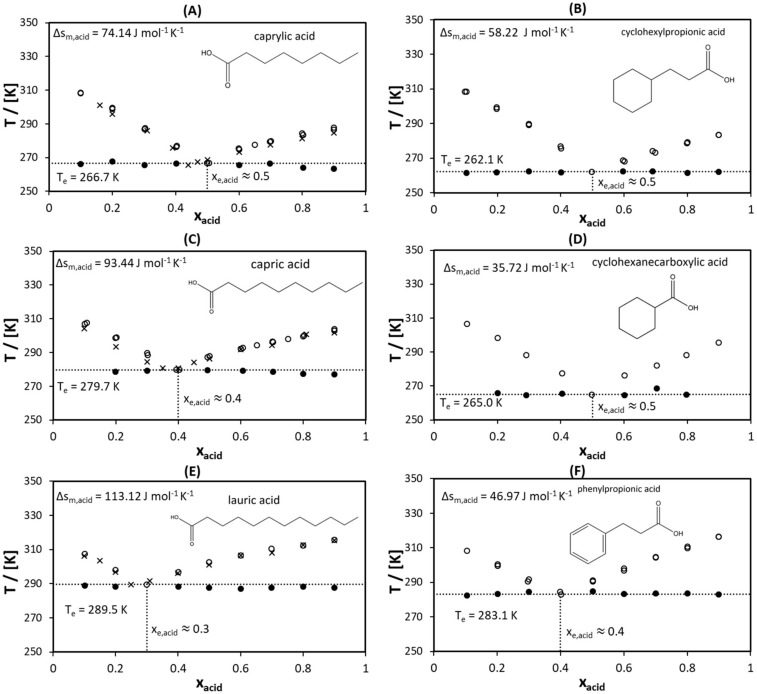
Solid–liquid phase diagrams of binary eutectic mixtures consisting of l-menthol and (**A**) caprylic acid, (**B**) cyclohexylpropionic acid, (**C**) capric acid, (**D**) cyclohexanecarboxylic acid, (**E**) lauric acid, and (**F**) phenylpropionic acid. The melting properties presented are experimentally determined values. Legend: ◦ liquidus temperature measured in this study, • the experimental eutectic temperature measured in this study, × Martins et al. [49].

To validate the proposed approach, the SLE of the six eutectic systems was measured using DSC. Figure 2 presents the measured SLE data for l-menthol with six different monocarboxylic acids. To simplify the comparison between systems, the systems containing acids of similar melting temperatures are presented next to each other (Figure 2A–F). The approximated melting temperature of acids in each pair increased from Figure 2A,B (≈ 15 °C) to Figure 2E,F (≈ 45 °C). Martins et al. [49] measured the SLE of binary eutectic mixtures of L-menthol with caprylic acid, capric acid, and lauric acid. As seen from Figure 2A,C,E, the determined eutectic temperatures were in good agreement with the results reported by Martins et al. [49]. The slightly higher liquidus temperatures measured in this study might be due to the greater heating rate; in this study, a heating rate of 5 K min^–1^ was used, while in Martins et al. [49], 1 K min^–1^ was used.

As demonstrated in previous studies [35,36,37,38], the lower the melting enthalpy of the pure components, the higher the depression at the eutectic point. This could be confirmed by comparing the eutectic temperatures of eutectic systems formed between l-menthol and acids presented in Figure 2. The molecules with cyclic structures (Figure 2B,D,F) had lower flexibility than molecules with linear structures (Figure 2A,C,E). Therefore, the cyclic compounds possessed lower melting entropies. Because the melting temperatures of each pair of acids were similar (caprylic acid and cyclohexylpropionic acid ≈ 15 °C, capric acid and cyclohexanecarboxylic acid ≈ 30 °C, and lauric acid and phenylpropionic acid ≈ 45 °C), the melting enthalpy of cyclic compounds was lower than that of linear ones. As a result, the eutectic temperature of a system formed by a cyclic acid was lower than that of a system formed by a linear acid when both acids had the same melting temperature.

As seen in Figure 2, as the difference in the melting entropy of acids increased, the difference in the eutectic temperature increased. For example, the melting entropy of capric acid was almost three times higher than that of cyclohexanecarboxylic acid (see Figure 2C,D). This resulted in a eutectic temperature for l-menthol/cyclohexanecarboxylic acid (Figure 2C), which was approximately 15 K lower than that of l-menthol/capric acid (Figure 2D). For l-menthol/caprylic acid (Figure 2A) and l-menthol/3-cyclohexylpropionic acid (Figure 2B), the difference between their eutectic temperatures was only 4.5 K. This might be the result of a small difference between the melting entropies of caprylic acid and 3-cyclohexylpropionic acid (see Figure 2A,B). In a previous study [38], it was demonstrated that the eutectic composition was shifted toward the component that had a lower melting enthalpy. Comparing the eutectic composition between the systems revealed that the lower the melting enthalpy compared to that of l-menthol (13.74 kJ mol^−1^), the higher the mole fraction of the acid at the eutectic point.

It could be concluded that a lower melting enthalpy of pure components led to a larger melting temperature depression at the eutectic point of all l-menthol/monocarboxylic acid systems studied in this work. The melting enthalpy of components could be correlated with the molecular structure of acids possessing the same melting temperature. Therefore, the eutectic systems formed between l-menthol and cyclic acids exhibited a deeper eutectic point than that of systems formed with linear acids. Because the depression at the eutectic point was related to the difference in the melting entropy of the pure components, the relative depression at the eutectic point between the systems could also be predicted. Thus, the approach of selecting components by assessing the flexibility of their molecular structures could be used to design deeper eutectic systems.

## 3. Materials and Methods

### 3.1. Prediction of Melting Entropy

The melting entropy of the pure components was calculated as the sum of the rotational $∆s_{m}^{rot}$, conformational $∆s_{m}^{conf}$, and expansional entropies $∆s_{m}^{expan}$ as follows [41]:

(3) $$∆s_{m}=\;W\;+∆s_{m}^{rot}\;+\;∆s_{m}^{conf}\;+\;∆s_{m}^{expan}$$

where W is a constant. In this study, the model proposed by Jain et al. [44] was used to predict the melting entropy of the pure components. The melting entropy in J mol^–1^ K^–1^ was calculated as follows [44]:

(4) $$∆s_{m}=\;50\;+\;∆s_{m}^{rot}\;+\;∆s_{m}^{conf}$$

(5) $$∆s_{m}^{rot}=-\;R\ln\sigma$$

(6) $$∆s_{m}^{conf}=\;R\ln\Phi$$

where σ is the symmetry number, Φ is the flexibility number, and R is the universal gas constant. Readers are directed to the original paper [44] for more information about the determination of the symmetry number σ of the components. The flexibility number Φ was calculated as follows [44]:

(7) $$\Phi =\;2.435^{\tau }$$

(8) $$\tau =\;SP^{3}\;+\;\;0.5SP^{2}\;+\;0.5Ring\;-1$$

where SP^3^ is the number of non-ring SP^3^ atoms (CH_2_, CH, C, NH, N, O, S), SP^2^ is the number of SP^2^ atoms (=CH,=C,=N, C=O), and Ring is the number of independent single, fused, or conjugated ring systems. If τ is less than zero, the flexibility number Φ is set to 1.

In the search for components with low melting enthalpy values, components with low melting entropy were sought. According to Equations (4)–(6), components with symmetrical (large symmetry number σ) and/or more rigid molecular structures (small flexibility number Φ) should possess lower melting entropy. According to Equations (7) and (8), components with ring systems and double bonds should have a lower flexibility number Φ than single bond chains.

### 3.2. Eutectic Mixture Constituents

Binary eutectic mixtures of l-menthol with six different monocarboxylic acids were considered. The acids were sorted into three pairs according to the melting temperatures reported by the suppliers. The acids of each pair had close melting temperatures. The acids pairs were as follows:

(i) Caprylic acid and 3-cyclohexylpropionic acid with a melting temperature of approximately 15 °C,
(ii) Capric acid and cyclohexanecarboxylic acid with a melting temperature of approximately 30 °C,
(iii) Lauric acid and 3-phenylpropionic acid with a melting temperature of approximately 45 °C.

**Figure 3:**
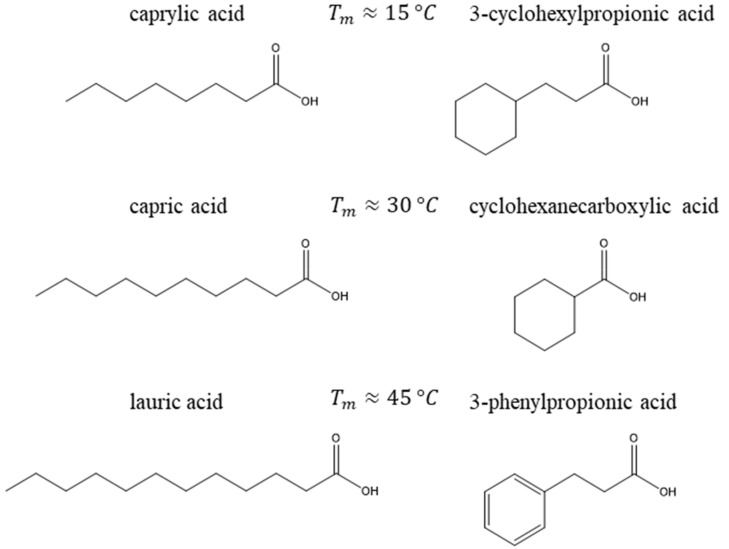
Molecular structures of monocarboxylic acids considered in this study. The melting temperatures are approximate values reported by the suppliers used to select each pair of acids.

In each pair, the acid with a more rigid molecular structure was expected to have lower melting entropy, and according to Equation (2), lower melting enthalpy. Therefore, the eutectic systems formed between l-menthol and rigid acids were expected to have a deeper eutectic temperature as a result of their lower melting enthalpy. Figure 3 illustrates the differences in the molecular structures of the acids, paired based on the melting temperatures obtained from the suppliers.

### 3.3. Eutectic Mixture Preparation

Table 4 lists the chemicals used in this study, along with their purity, as declared by the suppliers. All chemicals were used as received without further purification. The binary eutectic mixtures were prepared by weighing the pure components in a glass vessel. The mixture was heated to 45 °C while stirring with a magnetic stirrer until a homogenous liquid was obtained.

The liquid samples were weighed in aluminum DSC crucibles using a syringe and then sealed by cold welding. Depending on the density of the eutectic mixture, the mass of the samples in the crucibles ranged between 4 and 6 mg.

### 3.4. Differential Scanning Calorimetry

SLE data and the pure components’ melting properties were measured using DSC (NETZSCH DSC 200 F3). Temperature and sensitivity calibrations were performed prior to the measurements using five calibration standards with a purity of over 99.999%. The calibration standards included adamantane, bismuth, indium, zinc, and tin. The uncertainties after the temperature and sensitivity calibration were < 0.1 K and < 3%, respectively.

The DSC measurements were performed in an inert nitrogen environment. First, a cooling cycle with a rate of 10 K min^−1^ to a final temperature of −80 °C was performed. Then, the sample was heated at a heating rate of 5 K min^–1^ up to approximately 10 K above the sample liquidus temperature. The eutectic temperature, as well as the pure components’ melting temperature, were determined as the onset temperature of the respective thermal event. The liquidus temperatures were determined as the peak temperature, and the melting enthalpy of the pure components was determined as the peak area of the respective thermal event. The DSC curves of the samples obtained during the heating cycle are shown in Figures S2–S4 in Supplementary Materials.

## 4. Conclusions

In this study, a simple approach was proposed to select constituents for eutectic systems. This approach could be used to select constituents from a pool of substances sharing the same functionality and melting temperature based on their melting enthalpy. If the melting enthalpy is not available, it can be estimated from the melting entropy, which is correlated with the molecular structure, using the simple non-group contribution model proposed by Jain et al. [44].

The proposed approach was used to predict the relative depression of the melting temperature at the eutectic point of l-menthol/monocarboxylic acid systems. It was demonstrated that components with more rigid molecular structures possessed lower melting entropy. For linear and cyclic acids with similar melting temperatures, the cyclic acids possessed lower melting enthalpy due to their lower melting entropy. As a result, deeper eutectic systems could be formed by cyclic acids than by linear acids sharing the same melting temperature. Furthermore, the larger the difference in the melting entropy between the acids in each pair, the higher the relative depression at the eutectic point between the two eutectic systems.

From this study, it could be concluded that in the search for new DES systems, constituents with more rigid and symmetrical structures should be pursued. This could narrow the pool of possible components to be screened for a specific application based on eutectic temperature (i.e., when eutectic mixtures that are liquid at room temperature are investigated). It should be mentioned, however, that for quantitative predictions, experimental melting properties should be used along with activity coefficient modeling, using, for example, excess Gibbs energy models (g^E^) or equation of states.

## Figures and Tables

**Table 1:** Symmetry number σ, flexibility number Φ, and calculated melting entropy Δs_m_ of components using the model proposed by Jain et al. [44].

Compound	σ	SP3	SP2	Ring	τ	Φ	Δs_m_ (J mol^–1^ K^–1^)
3-cyclohexylpropionic acid	1	2	1	1	2	5.93	64.80
caprylic acid	1	6	1	0	5.5	133.58	90.69
cyclohexanecarboxylic acid	1	0	1	1	0	1	50
capric acid	1	8	1	0	7.5	792.03	105.49
3-phenylpropionic acid	1	2	1	1	2	5.93	64.80
lauric acid	1	10	1	0	9.5	4696.13	120.29

**Table 2:** Comparison of melting enthalpies Δh_m_ and temperatures T_m_ measured in this study and reported in the literature.

Compound	T_m_ (K)		Δh_m_ (kJ mol^–1^)	
	This Work *	Lit.	This Work *	Lit.
l-menthol	314.6 ± 0.1	315.68 [49]	13.74 ± 0.5	12.89 [49]
3-cyclohexylpropionic acid	291.3 ± 0.1	–	16.96 ± 0.5	–
caprylic acid	288.0 ± 0.7	288.20 [49]	21.43 ± 0.3	19.80 [49]
cyclohexanecarboxylic acid	299.4 ± 1.1	301.9 [50]	10.69 ± 0.2	9.20 [50]
capric acid	303.9 ± 0.1	304.75 [51]	28.39 ± 0.7	27.50 [51]
3-phenylpropionic acid	321.6 ± 0.1	321.2 [52]	15.11 ± 0.1	15.68 [52]
lauric acid	316.6 ± 0.1	317.48 [51]	35.81 ± 0.4	37.83 [51]

* Uncertainties are considered as the standard deviation of three measurements.

**Table 3:** Comparison of predicted Δs_m_^predicted^ and experimental Δs_m_^experimental^ melting entropy of monocarboxylic acids.

Compound	Δs_m_^predicted^	Δs_m_^experimental^
3-cyclohexylpropionic acid	64.80	58.22
caprylic acid	90.69	74.41
cyclohexanecarboxylic acid	50	35.72
capric acid	105.49	93.44
3-phenylpropionic acid	64.80	46.97
lauric acid	120.29	113.12

**Table 4:** Chemicals used in this study.

Name	CAS Number	Supplier	Purity *
l-menthol	2216–51–5	Sigma Aldrich Chemie GmbH	≥ 99 %
3-cyclohexylpropionic acid	701–97–3	ThermoFisher (Kandel) GmbH	> 98 %
caprylic acid	124–07–2	Merck KGaA	99 %
cyclohexanecarboxylic acid	98–89–5	ThermoFisher (Kandel) GmbH	98 %
capric acid	334–48–5	Alfa Aesar GmbH	99 %
3-phenylpropionic acid	501–52–0	Alfa Aesar GmbH	99 %
lauric acid	143–07–7	Merck KGaA	99 %

* As declared by the supplier.

## Supplementary Materials

The following are available online. Figure S1. Solid-liquid phase diagrams of binary eutectic systems consisting of l-menthol and (**A**) cyclohexylpropionic acid (**B**) caprylic acid (**C**) cyclohexanecarboxylic acid (**D**) capric acid (**E**) phenylpropionic acid (**F**) lauric acid. Dashed lines are ideal liquidus lines of components modeled using the Schöder-van-Laar equation and using experimental melting properties. Figure S2. DSC curves of l-menthol/3-cyclohexylpropionic acid and l-menthol/caprylic acid systems. Figure S3. DSC curves of l-menthol/cyclohexanecarboxylic acid and l-menthol/capric acid systems. Figure S4. DSC curves of l-menthol/3-phenylpropionic acid and l-menthol/lauric acid systems.
