# Supplementary material for: Design of Deep Eutectic Systems: A Simple Approach for Preselecting Eutectic Mixture Constituents
Source: Molecules. 2020 Feb 28;25(5):1077. doi: 10.3390/molecules25051077 (PMC7179121; doi:10.3390/molecules25051077)
Supplement: Supplementary file 1 [file molecules-25-01077-s001.pdf]

Supplementary Material

Design of Deep Eutectic Systems: A Simple Approach for Preselecting Eutectic Mixture Constituents

Ahmad Alhadid [1], Liudmila Mokrushina [2] and Mirjana Minceva [1,*]

[1] Technical University of Munich, Biothermodynamics, TUM School of Life and Food Sciences Weihenstephan, Maximus-von-Imhof-Forum 2, 85354 Freising, Germany; ahmad.alhadid@tum.de; mirjana.minceva@tum.de

[2] Friedrich-Alexander-Universität Erlangen-Nürnberg (FAU), Separation Science & Technology, Egerlandstr. 3, 91058 Erlangen, Germany; liudmila.mokrushina@fau.de.

* Correspondence: mirjana.minceva@tum.de

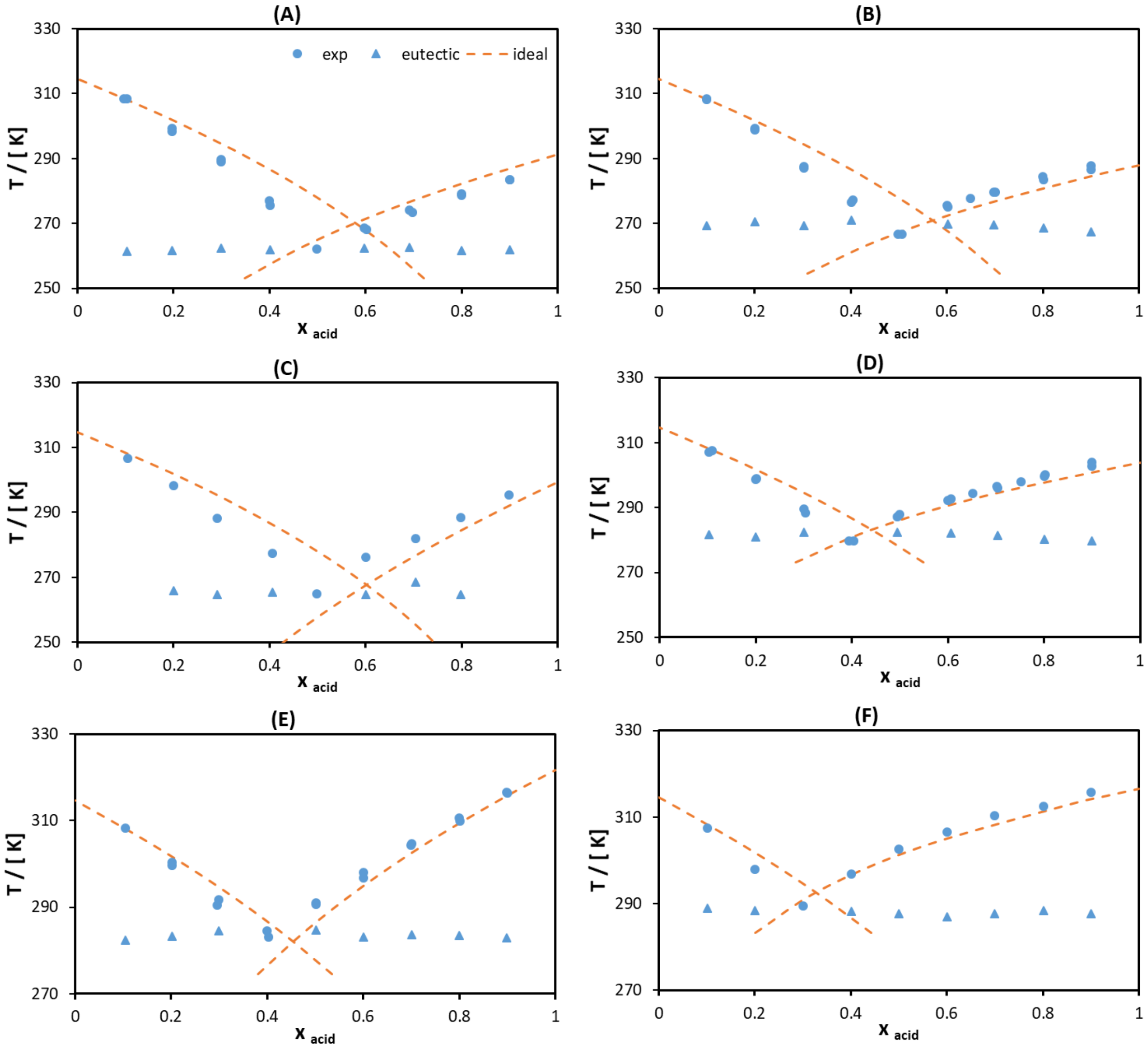

Figure S1. Solid–liquid phase diagrams of binary eutectic systems consist of L-menthol and (A) cyclohexylpropionic acid (B) caprylic acid (C) cyclohexanecarboxylic acid (D) capric acid (E) phenylpropionic acid (F) lauric acid. Dashed lines are ideal liquidus lines of components modeled using the Schöder–van–Laar equation and using experimental melting properties.

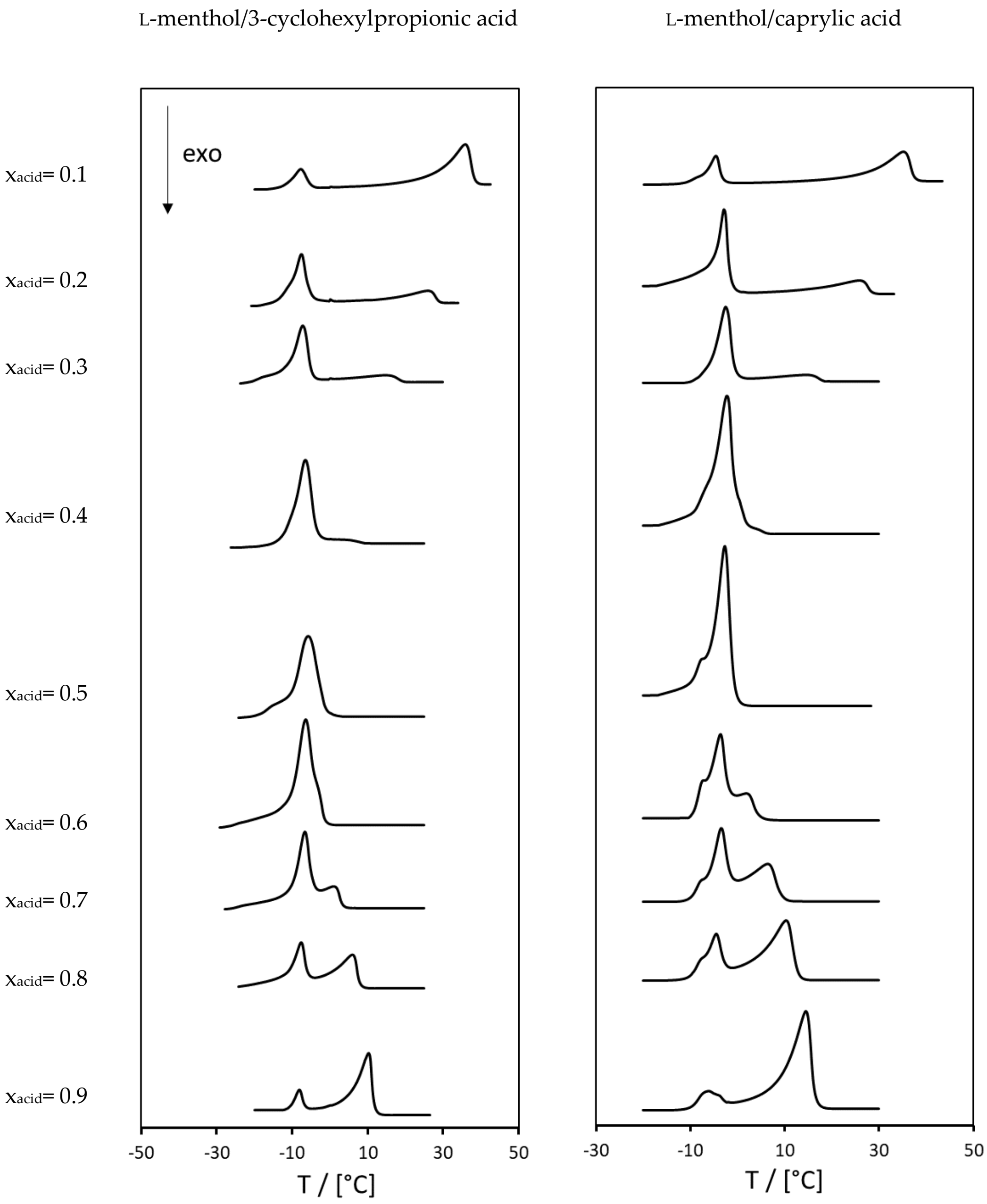

Figure S2. DSC curves of L-menthol/3-cyclohexylpropionic acid and L-menthol/caprylic acid systems

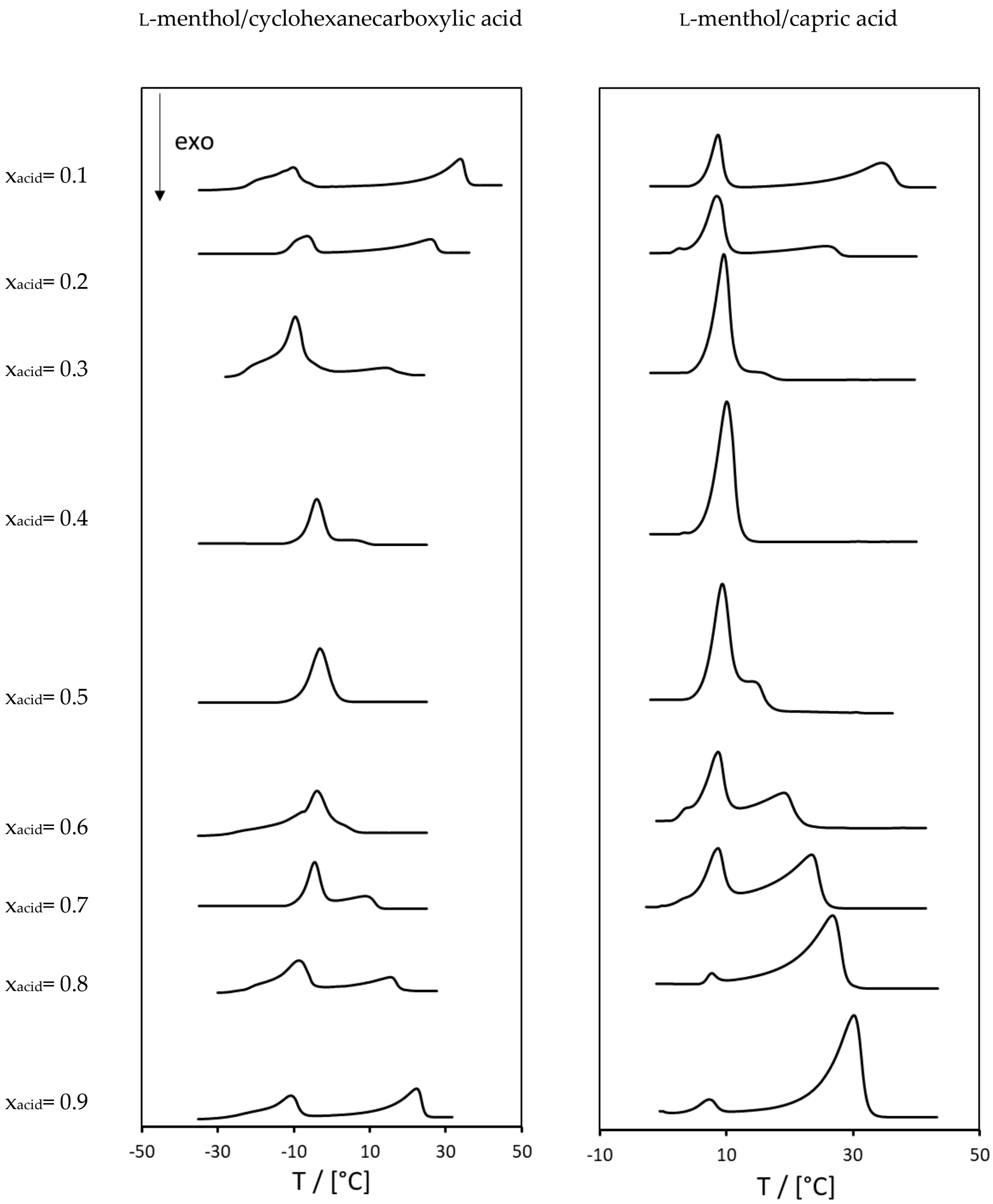

Figure S3. DSC curves of L-menthol/cyclohexanecarboxylic acid and L-menthol/capric acid systems

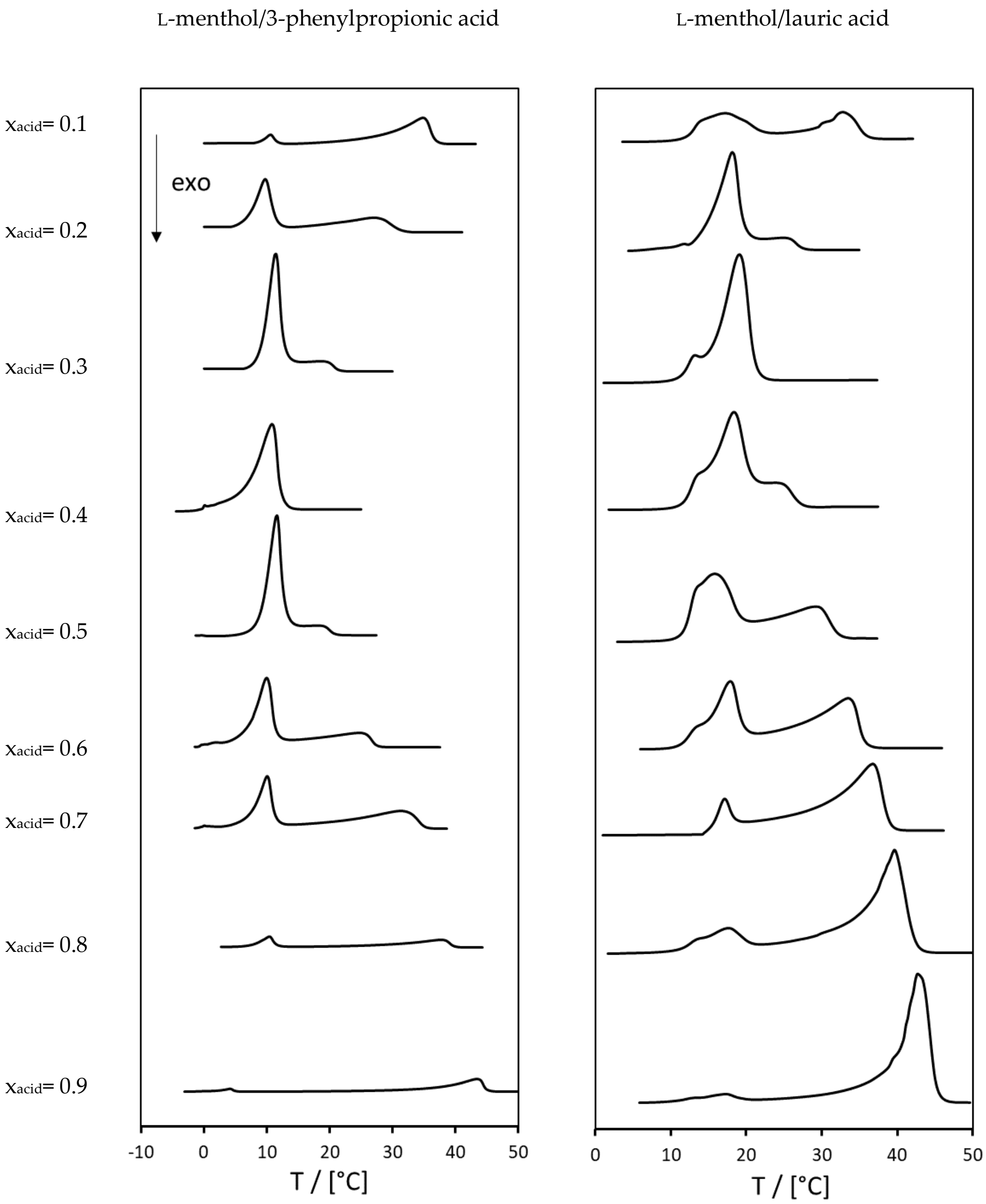

Figure S4. DSC curves of L-menthol/3-phenylpropionic acid and L-menthol/lauric acid systems
